# Retroperitoneal Teratoma in a Newborn: A Case Report and Diagnostic Insights

**DOI:** 10.7759/cureus.75450

**Published:** 2024-12-10

**Authors:** Radhika Maddali, Palanikumar Balasundaram, Benjamin A Farber, Mariam S LaTuga

**Affiliations:** 1 Department of Pediatrics, Division of Neonatology, The Children’s Hospital at Montefiore and Albert Einstein College of Medicine, Bronx, USA; 2 Department of Pediatrics, Division of Neonatology, Mercy Health, Javon Bea Hospital, University of Illinois College Medicine of Rockford, Rockford, USA; 3 Department of Pediatrics Surgery, Loma Linda University Children's Hospital, Loma Linda, USA; 4 Department of Pediatrics, Division of Neonatology, Blythedale Children's Hospital, Valhalla, USA

**Keywords:** congenital tumor, immature teratoma, neonatal abdominal mass, neonate, retroperitoneal teratoma

## Abstract

Retroperitoneal teratomas are rare neoplasms in neonates, presenting with nonspecific symptoms and variable clinical features, making diagnosis challenging. Radiological investigations, particularly fetal ultrasound and contrast-enhanced computed tomography, play a critical role in their detection. Differential diagnoses include neuroblastoma, adrenal hemorrhage, and congenital cystic lesions, which share overlapping clinical and imaging features. This case report describes a neonate delivered at 39 weeks of gestation, weighing 2.93 kg, to a 30-year-old gravida 2, para 1 mother with unremarkable serological tests during pregnancy. Fetal ultrasonography performed at 31 weeks, followed by magnetic resonance imaging at 35 weeks, revealed a large, multi-cystic, and solid lesion located above the left kidney. Postnatal physical examination identified a firm, non-tender abdominal mass that was confined to the left side and did not cross the midline. Imaging studies, including abdominal radiographs, ultrasonography, and contrast-enhanced computed tomography, confirmed a large left supra-renal mass. Meta-iodo-benzyl-guanidine scintigraphy combined with single-photon emission computed tomography showed no activity in the mass. Serum alpha-fetoprotein levels were within the normal range for term neonates, and the infant had normal beta-human chorionic gonadotropin and urine homovanillic acid levels. The infant underwent exploratory laparotomy on the sixth postnatal day, confirming an immature teratoma without malignant components. Postoperatively, AFP levels demonstrated the expected physiological decline, consistent with the absence of malignant components. The infant was discharged on postnatal day 35. This case highlights the diagnostic complexities of retroperitoneal teratomas in neonates and underscores the critical role of antenatal ultrasound and a multidisciplinary approach in ensuring effective diagnosis and management.

## Introduction

Retroperitoneal teratoma (RPT) is a rare tumor derived from primitive germ cells, containing mixed dermal elements originating from all three germ layers-ectoderm, endoderm, and mesoderm. These tumors are primarily found in neonates and young adults [1]. In the pediatric population, RPTs rank as the third most common primary retroperitoneal tumors, following neuroblastoma and Wilms tumor [2]. RPTs represent 2%-5% of all teratomas and are exceptionally rare in the neonatal period [1]. RPTs exhibit a bimodal age distribution, peaking in infancy and young adulthood [3]. In the neonatal period, they are rare but can be identified antenatally through routine fetal imaging or postnatally due to abdominal distension or palpable masses. Although they are generally benign and slowly developing, their nonspecific symptoms and varied presentations pose diagnostic challenges. A comprehensive diagnostic approach that integrates advanced imaging techniques with histopathological evaluation is highly effective in diagnosing RPTs. Advancements in imaging techniques such as fetal MRI and contrast-enhanced CT have significantly improved the diagnostic accuracy of RPTs by providing high-resolution details about the tumor's composition, vascularity, and relationship to adjacent structures. In addition, innovations in histopathological analysis, including immunohistochemical markers and molecular profiling, can further enhance diagnostic precision and help differentiate RPTs from other retroperitoneal masses.

## Case presentation

**Figure 1:**
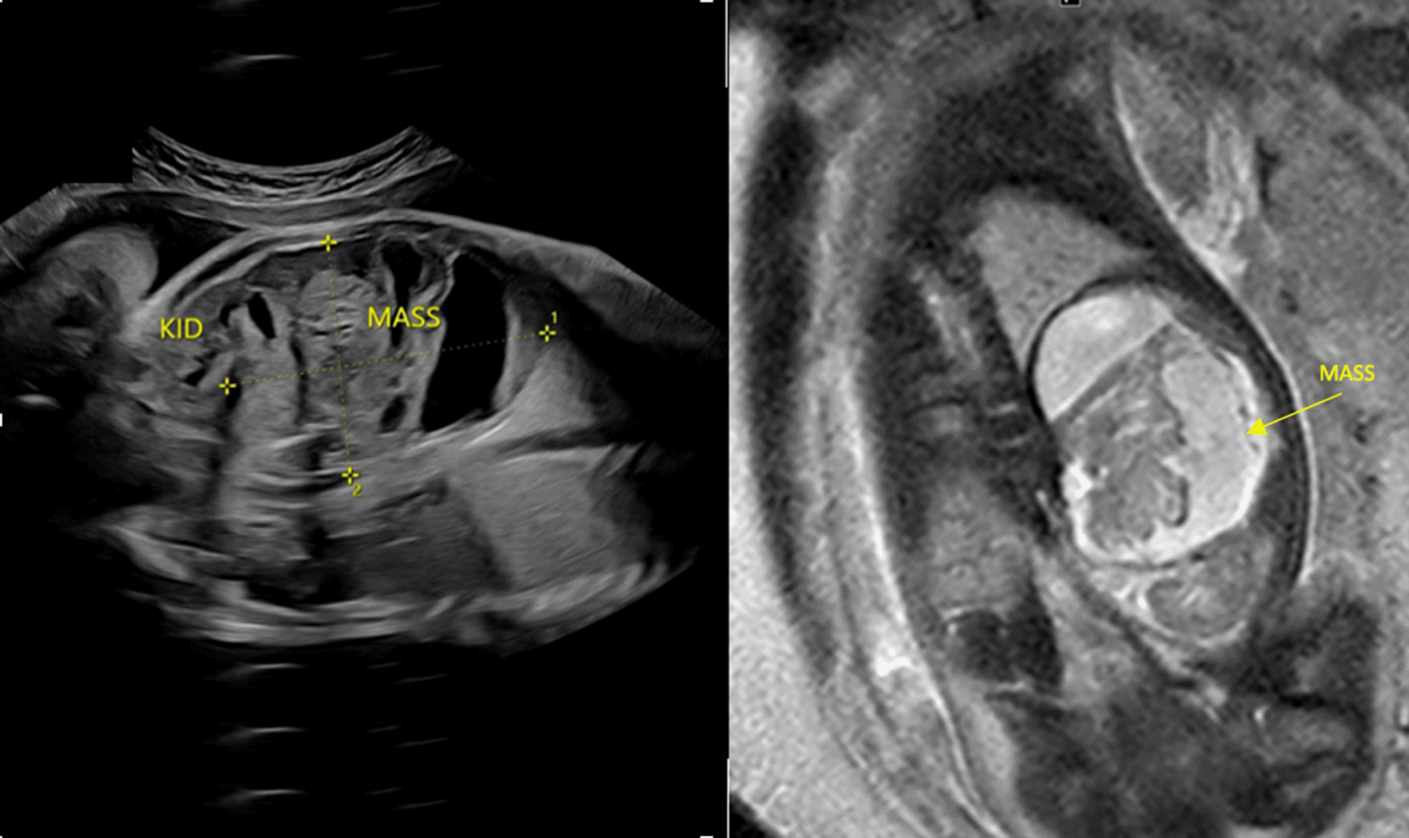
Fetal ultrasound (left) and fetal MRI (right) showing a large left suprarenal multi-cystic and solid lesion.

A 2.93 kg female neonate was delivered vaginally at 39 weeks of gestation to a 30-year-old gravida 2, para 1 woman. Fetal ultrasonography (USG) at 31 weeks gestation revealed a cystic structure in the left abdominal region. Further evaluation with fetal magnetic resonance imaging (MRI) at 35 weeks identified a large, multi-cystic, solid lesion located above the left kidney (Figure 1).

**Figure 2:**
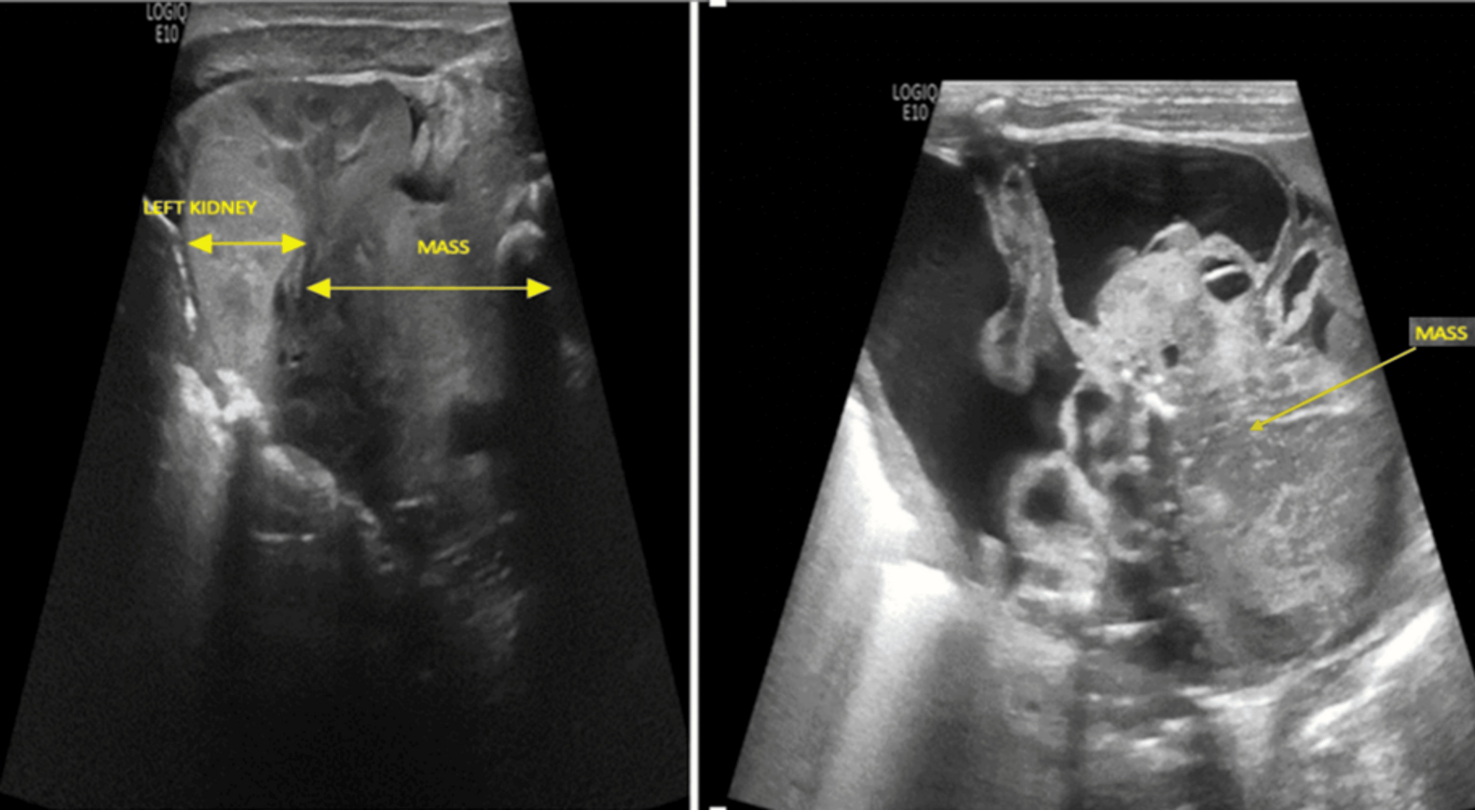
(Left, right) Abdominal ultrasound images revealing a large mass located above the left kidney, comprising multiple cystic and solid components.

The neonate presented with an abdominal mass and mild abdominal distension without additional symptoms. Physical examination revealed a firm, non-tender mass palpable on the left side of the abdomen, with the neonate exhibiting normal vital signs. An abdominal radiograph demonstrated a large, calcified mass in the left abdomen, causing displacement of bowel loops to the right. Abdominal USG (Figure 2) identified a left-sided suprarenal mass with internal Doppler flow.

**Figure 3:**
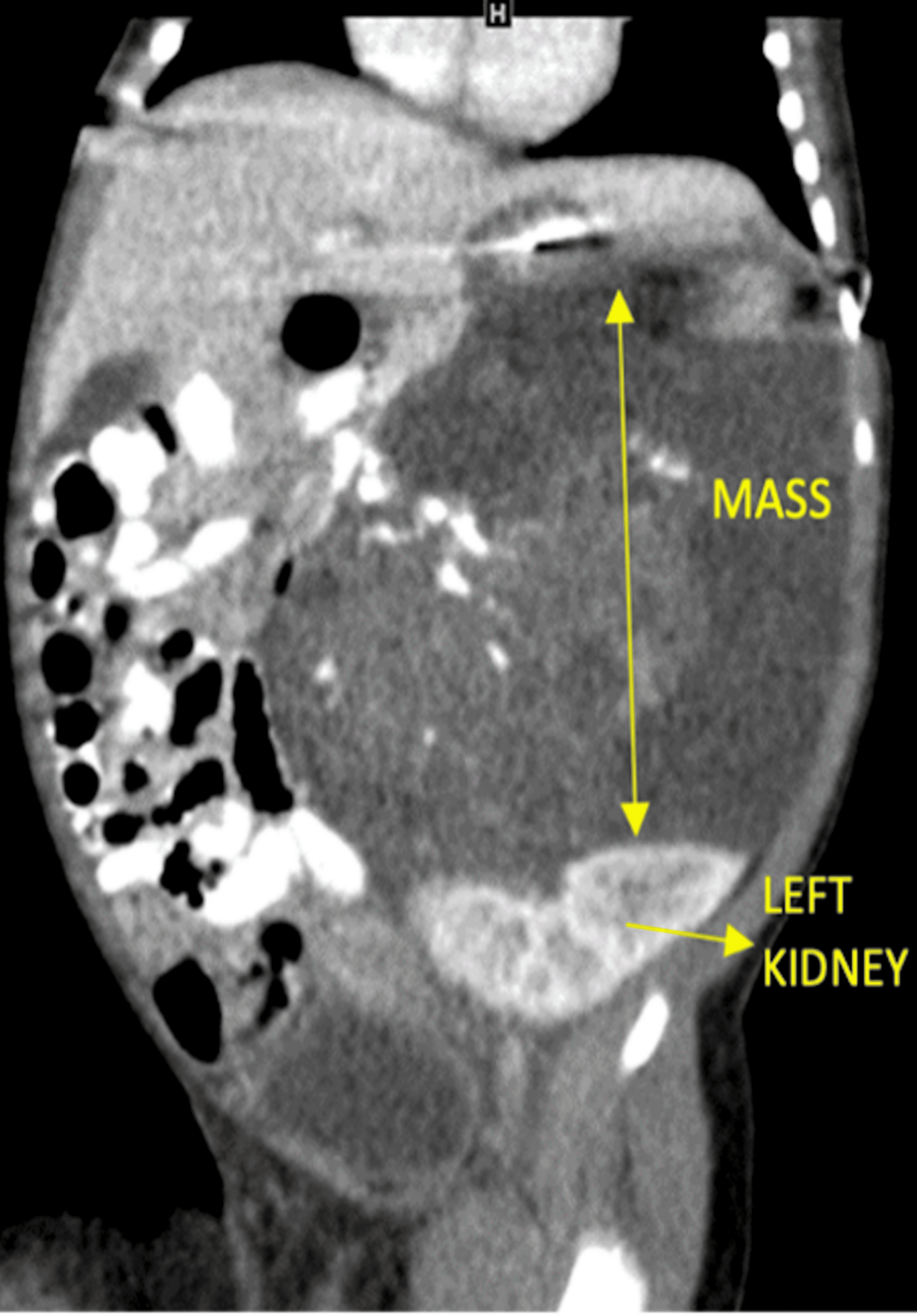
Abdominal CECT revealing a large mass superior to the left kidney with a combination of cystic and soft tissue components, calcification, and an absence of internal fat.

A contrast-enhanced computed tomography (CECT) scan (Figure 3) performed on the fourth day of life revealed a large left suprarenal mass with cystic and soft tissue components, calcifications, and no internal fat.

**Figure 4:**
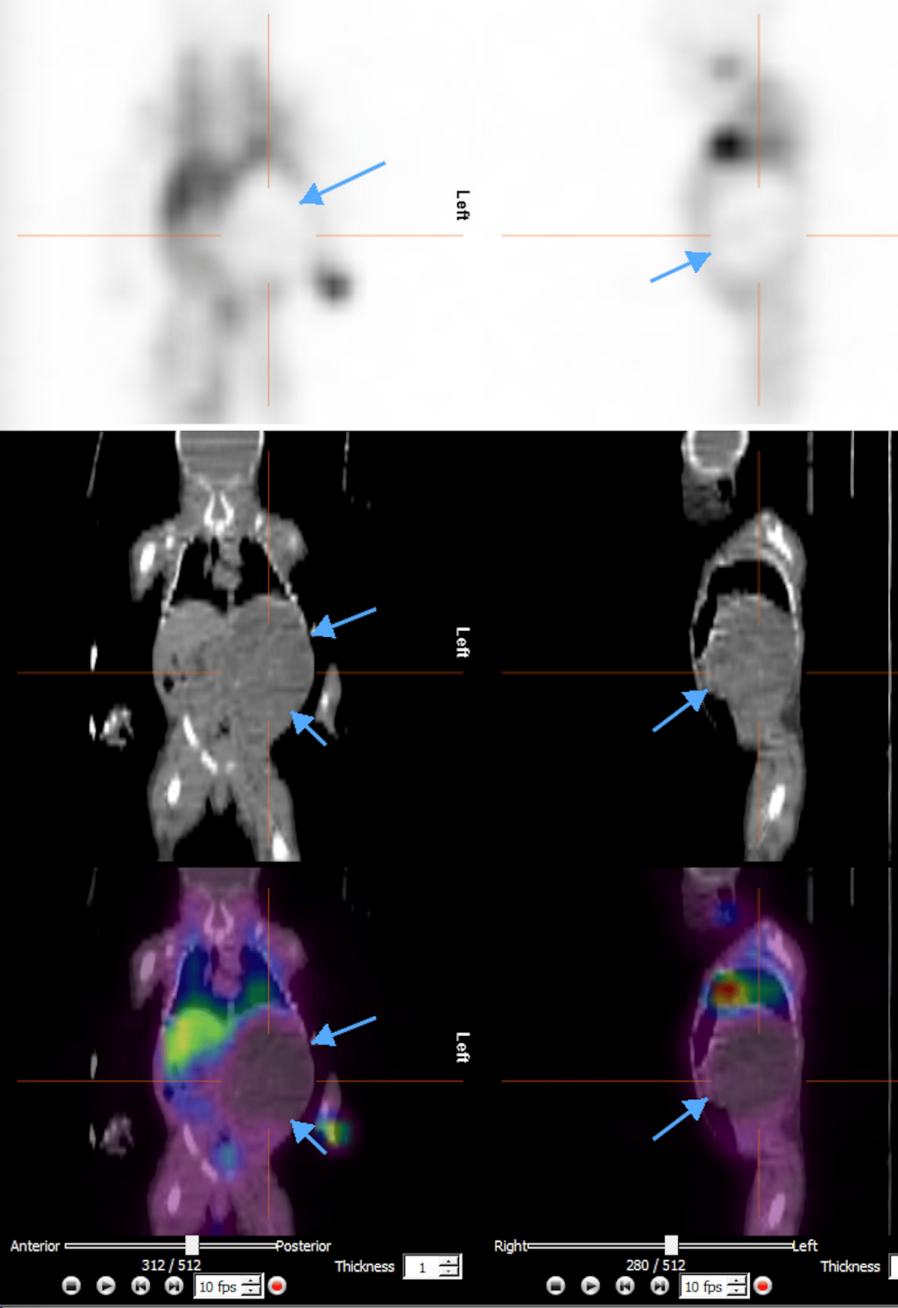
Meta-iodo-benzyl-guanidine (MIBG) scan showing no MIBG avidity within the intra-abdominal mass.

A meta-iodo-benzyl-guanidine (MIBG) scan showed no MIBG avidity within the intra-abdominal mass (Figure 4). Serum alpha-fetoprotein (AFP) was 95,343 ng/mL, falling within the expected range for term neonates (9,120-190,546 ng/mL). Additionally, serum beta-human chorionic gonadotropin (β-HCG) was < 6 mIU/mL, and urine homovanillic acid (HVA) was 8.3 mg/g creatinine (normal range: 0-42 mg/g creatinine for children under two years). Vanillylmandelic acid (VMA) levels were insufficient for analysis.

**Figure 5:**
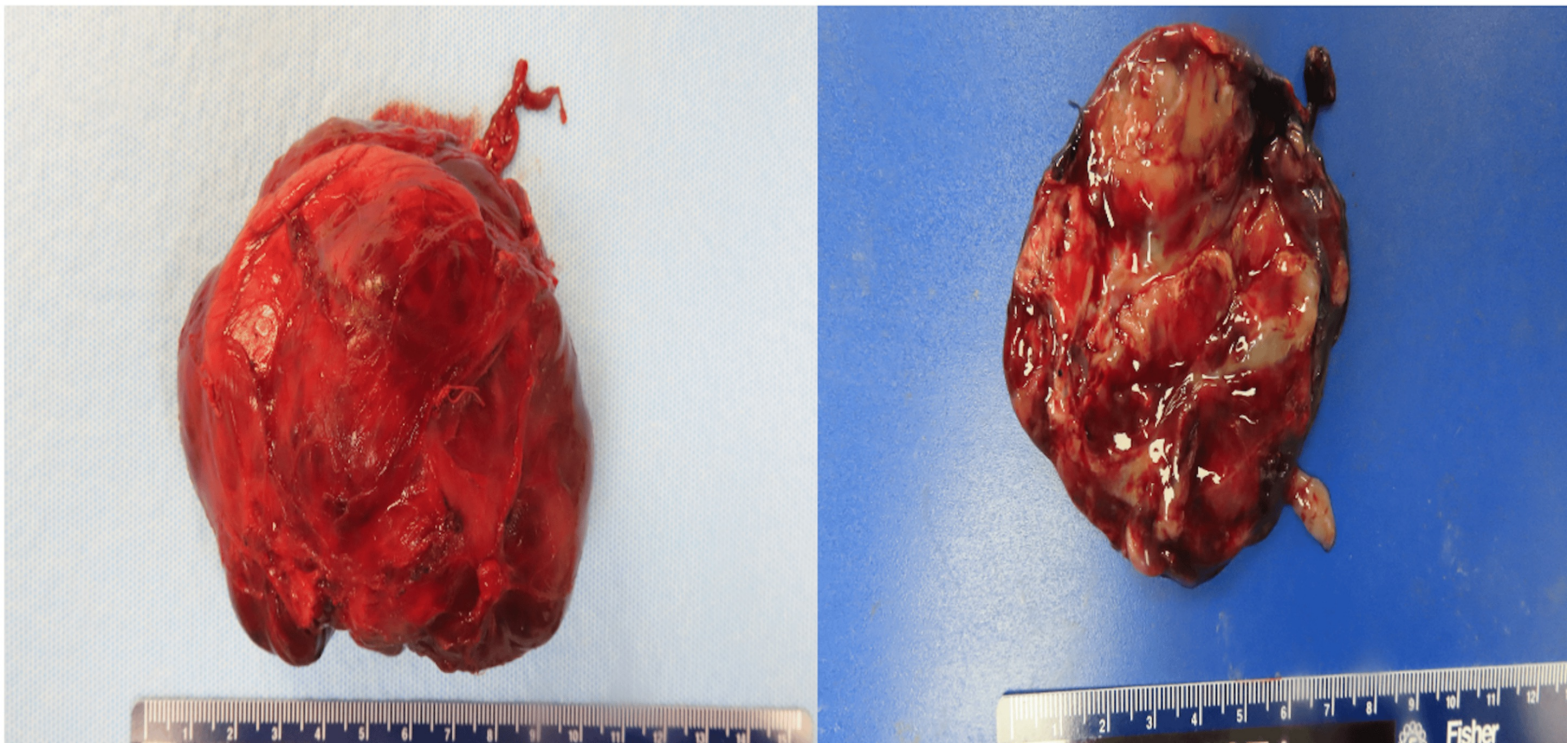
(Left, right) Macroscopic examination of resected specimen

For this term infant presenting with a large left-sided intra-abdominal mass characterized by cystic and solid components with calcifications, the differential diagnosis included neuroblastoma, retroperitoneal teratoma, and adrenal hemorrhage. With both cystic and solid elements, the mass's complexity posed challenges for precise characterization through postnatal imaging. Upon reviewing the differential diagnosis, the presence of internal Doppler flow and the absence of a multiloculated cystic adrenal mass made adrenal hemorrhage less likely. While the well-defined cystic components and areas of calcification observed on CT imaging were consistent with features of a teratoma, the lack of internal fat was atypical for this diagnosis. Additionally, a negative MIBG scan did not definitively exclude neuroblastoma. Due to the persistent diagnostic uncertainty, surgical intervention was deemed necessary. On the sixth postnatal day, the left-sided retroperitoneal mass was successfully resected through an exploratory laparotomy. The encapsulated specimen measured 8.6 × 6.1 × 6.0 cm and exhibited a solid, nodular appearance with cystic areas and an intact capsule (Figure 5).

**Figure 6:**
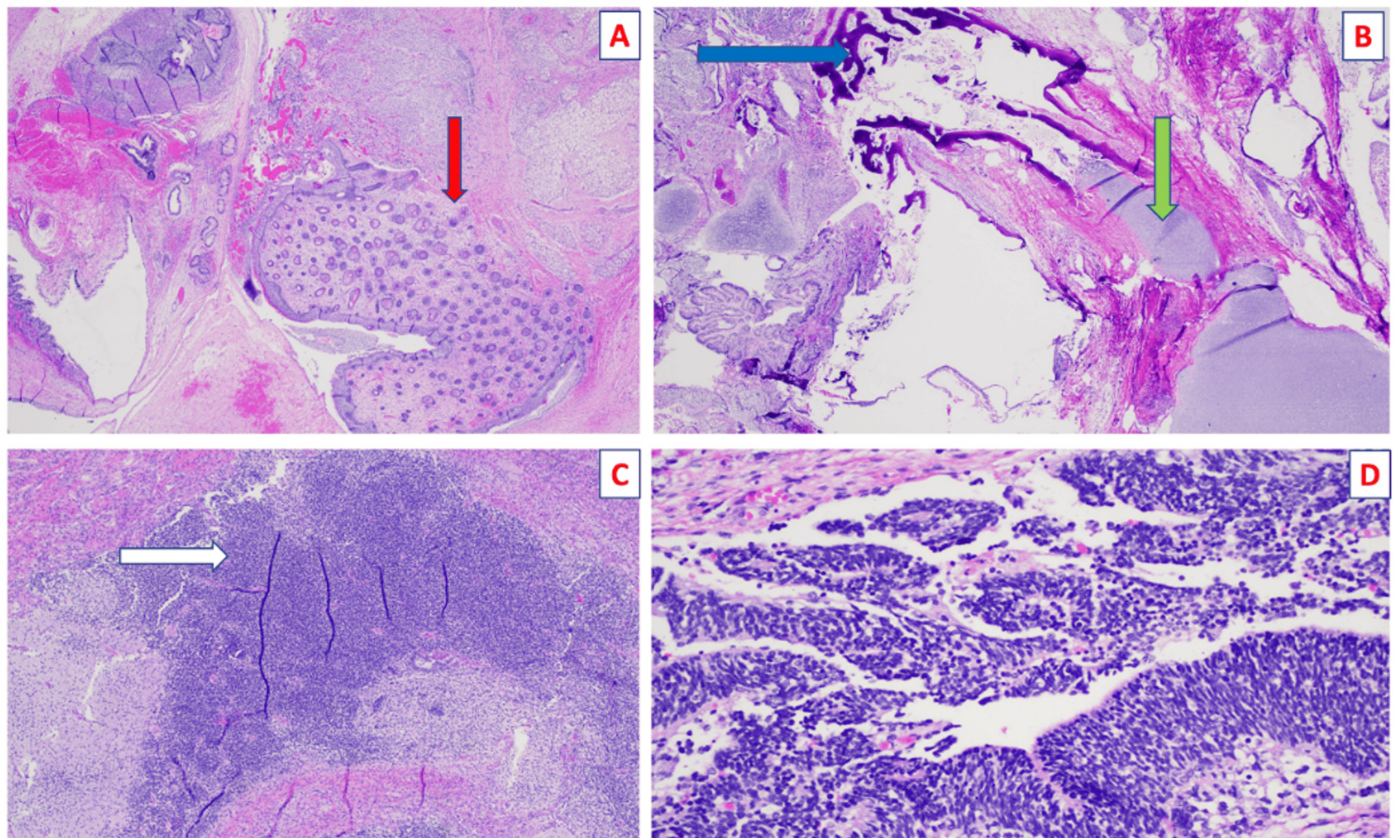
Microscopic examination of the resected specimen revealing the presence of skin with adnexal structures (red arrow in A), bone (blue arrow), and cartilage (green arrow) regions (B), as well as a notable presence of immature neuroectoderm (C; white arrow, D).

Microscopic examination (Figures 6A-6D) confirmed the presence of tissues from all three embryonic layers, including both mature and immature components, consistent with an immature teratoma without malignant features. Postoperatively, AFP levels demonstrated the expected physiological decline, consistent with the absence of malignant components. The neonate exhibited no complications such as bowel obstruction, hemorrhage, or infection, which are known potential complications of retroperitoneal teratomas. The neonate was discharged on postnatal day 35.

## Discussion

RPTs are rare congenital tumors, representing approximately 2%-5% of all teratomas [1]. Among neonates, sacrococcygeal teratomas are the most common congenital tumors [2]. In contrast, RPTs exhibit a bimodal age distribution, primarily occurring during infancy and young adulthood, with a peak in the first decade of life [3,4]. These tumors are most commonly located near the upper pole of the kidney, with a predilection for the left side [5].

The clinical presentation of RPT varies depending on the patient’s age, tumor location, and tumor growth rate. In the perinatal period, rapidly growing RPTs often present as a palpable abdominal mass or are identified incidentally during antenatal fetal ultrasounds [5-7]. In contrast, slow-growing RPTs may present later in childhood with nonspecific symptoms, typically resulting from compression of adjacent retroperitoneal organs [1,4,5]. RPTs in neonates often manifest as palpable abdominal masses or incidental findings on antenatal imaging, reflecting rapid growth, whereas in older children, slower-growing tumors present with nonspecific symptoms due to organ compression. Prenatal diagnosis focuses on early detection via imaging, while postnatal evaluation uses imaging, tumor markers, and histopathology to guide management.

Prenatal imaging of RPT typically involves fetal ultrasound and MRI. Fetal MRI, with its superior soft tissue resolution, is particularly effective in determining the origin of the mass and its relationship to surrounding organs, making it an invaluable tool for diagnosing fetal RPT [8]. Close monitoring with frequent fetal ultrasounds is recommended to track tumor growth [9]. Postnatally, plain radiographs may reveal calcifications or the presence of bone and teeth within the tumor. USG is useful for distinguishing between cystic and solid components. Comprehensive preoperative evaluation is achieved through CT and MRI, which help assess the nature of the teratoma, its spatial relationship to adjacent structures, and the extent of the lesion. Features, such as homogeneity, cyst formation, fat density, and calcifications, are indicative of benign retroperitoneal tumors [10].

Serum AFP levels are often elevated in patients with RPTs, particularly in cases involving high-grade immature or malignant tumors [11]. Monitoring AFP levels is a valuable tool for diagnosis, evaluating treatment response, and detecting tumor recurrence [12]. However, interpreting AFP levels in infants can be challenging due to the naturally high physiological levels during this age [13]. In some cases of RPT, elevated levels of β-HCG, carcinoembryonic antigen, and carbohydrate antigen 19-9 have also been reported [3]. Notably, in our patient, AFP and β-HCG levels were within the normal range.

Histologically, RPTs are classified based on the types of tissues present within the tumor. These categories include mature teratomas, immature teratomas, teratomas with malignant transformation, and mono-dermal teratomas [14]. Immature teratomas are defined by the presence of less differentiated tissue, while mature teratomas consist of well-differentiated structures derived from germ cell layers [15]. Although both mature and immature RPTs are generally benign, a thorough histological examination is crucial, particularly when high-grade features are observed, as these may be associated with microscopic malignancy [16].

Complete surgical excision with subsequent histopathological evaluation remains the cornerstone for the definitive diagnosis and treatment of RPTs. In infants, surgical management can be particularly challenging due to adhesions to adjacent structures and the risk of vascular distortion [17]. Complete resection is associated with an excellent prognosis for benign tumors [18], and postoperative follow-up typically involves clinical examinations, ultrasound imaging, and monitoring of serum AFP levels. Ultrasound imaging is typically performed at one month, three months, and six months post-surgery, with more frequent follow-up at the provider’s discretion if recurrence is suspected. Serum AFP levels are also monitored at these intervals until they return to normal, with more frequent monitoring recommended if AFP remains elevated. Incomplete excision increases the risk of tumor recurrence. Chemotherapy is generally reserved for cases involving malignant RPT, incomplete resections, or recurrences [19]. Mature RPTs with predominantly neurogenic elements carry a significant risk of malignant transformation, while higher-grade immature teratomas are more prone to recurrence and malignant transformation [20]. Although malignant changes are rare, the likelihood of malignancy increases with larger tumor sizes.

## Conclusions

Diagnosing RPTs in infancy can be difficult due to their nonspecific symptoms and clinical latency. To facilitate early detection, clinicians should prioritize the routine use of antenatal ultrasound, enabling timely intervention and improving the outcome. This case report highlights the importance of a multidisciplinary, comprehensive diagnostic approach that integrates advanced imaging techniques with histopathological evaluation to accurately diagnose RPT and guide tailored interventions for optimal patient care.
